# Crystal structure of di­aqua­[5,10,15,20-tetra­kis­(4-bromo­phen­yl)porphyrinato-κ^4^ *N*]magnesium

**DOI:** 10.1107/S2056989015003722

**Published:** 2015-02-28

**Authors:** Nesrine Amiri, Soumaya Nasri, Thierry Roisnel, Gérard Simonneaux, Habib Nasri

**Affiliations:** a Département de Chimie, Faculté des Sciences de Monastir, Université de Monastir, Avenue de l’environnement, 5019 Monastir, Tunisia; b Centre de Diffractométrie X, Institut des Sciences Chimiques de Rennes, UMR 6226, CNRS–Université de Rennes 1, Campus de Beaulieu, 35042 Rennes Cedex, France; c Institute of Sciences Chimiques of Rennes, Ingénierie Chimique et Molécules pour le vivant, UMR 6226 CNRS, Campus de Beaulieu, 35042 Rennes Cedex, France

**Keywords:** crystal structure, magnesium porphyrin complex, O—H⋯Br hydrogen bonds, C—H⋯Br inter­actions, C—H⋯π inter­actions

## Abstract

The title compound, [Mg(C_44_H_24_Br_4_N_4_)(H_2_O)_2_] or [Mg(TBrPP)(H_2_O)_2_], where TBrPP is the 5,10,15,20-tetra­kis­(4-bromo­phen­yl)porphyrinato ligand, was obtained unintentionally as a by-product of the reaction of the [Mg(TBrPP)] complex with an excess of di­methyl­glyoxime in di­chloro­methane. The entire mol­ecule exhibits point group symmetry 4/*m*. In the asymmetric unit, except for two C atoms of the phenyl ring, all other atoms lie on special positions. The Mg^II^ atom is situated at a site with symmetry 4/*m*, while the N and the C atoms of the porphyrin macrocycle, as well as two C atoms of the phenyl ring and the Br atom lie in the mirror plane containing the porphyrin core. The H atoms of the axially bonded water mol­ecule are incompatible with the fourfold rotation axis and are disordered over two sites. In the crystal, mol­ecules are packed in rows along [001]. Weak inter­molecular C—H⋯π and C—H⋯Br inter­actions, as well as O—H⋯Br hydrogen bonds, stabilize the crystal packing.

## Related literature   

For general background to magnesium porphyrin species and their applications, see: Ghosh *et al.* (2010 ▸). For the synthesis of the [Mg^II^(TPP)(H_2_O)] complex (TPP is the 5,10,15,20-tetra­phenyl­porphyrinate ligand), see: Timkovich & Tulinsky (1969 ▸). In the Cambridge Structural Database (CSD, Version 5.35; Groom & Allen, 2014 ▸), there are six magnesium porphyrin structures with aqua ligands deposited. Four from these structures are mono­aqua species, derived from [Mg(TOMePP)(H_2_O)] (TOMePP is the 5,10,15,20-tetra­kis­(4-meth­oxy­phen­yl)porphyrinate ligand; Yang & Jacobson, 1991 ▸) and one is a di­aqua derivative, [Mg(TPP)(H_2_O)_2_]·(18-C-6) where 18-C-6 is 18-crown-6 ether (Ezzayani *et al.*, 2013 ▸). For the related porphyrin species [Mg(TPP)(4-pic)_2_] (4-pic = 4-picoline) and [Mg(TPP)(H_2_O)], see: McKee *et al.* (1984 ▸) and Ong *et al.* (1986 ▸), respectively. The H atom position of the aqua axial ligand was calculated with the program *CALC-OH* (Nardelli, 1999 ▸).
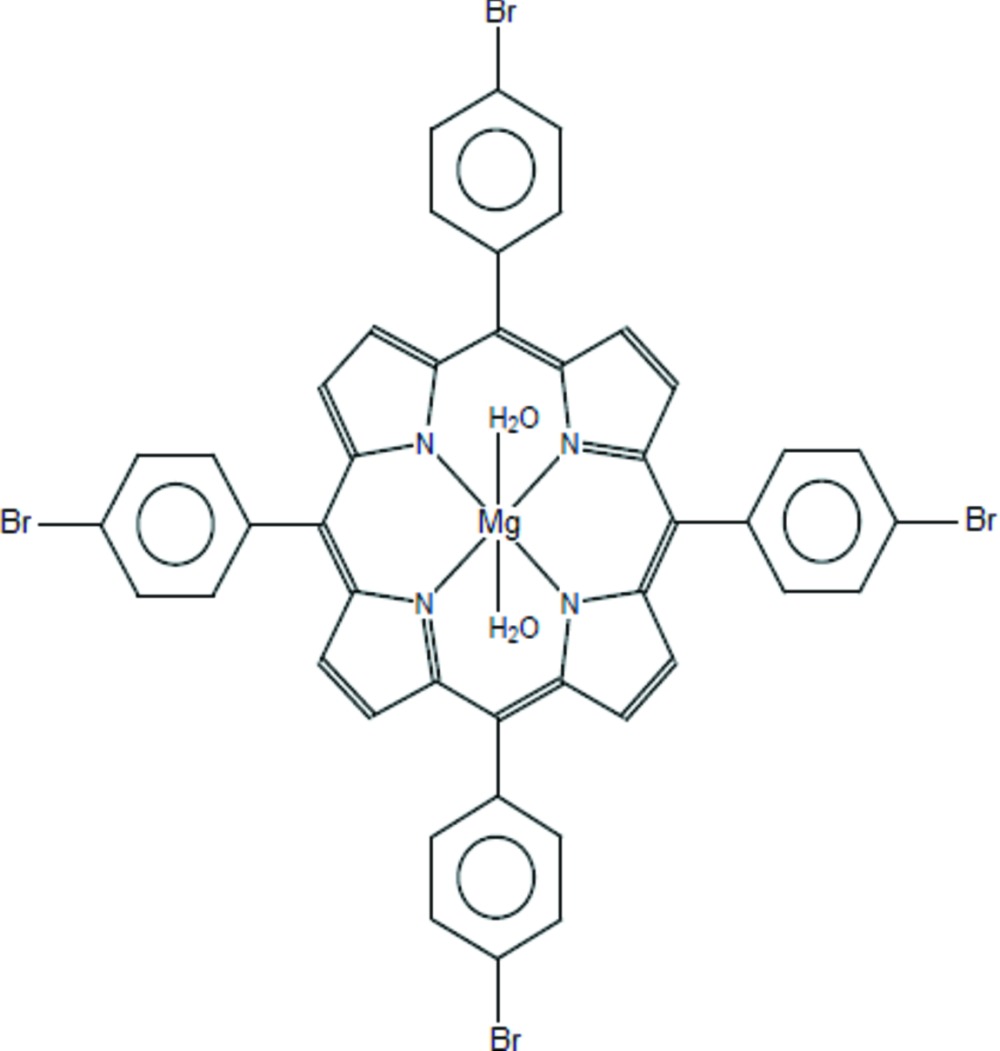


## Experimental   

### Crystal data  

[Mg(C_44_H_24_Br_4_N_4_)(H_2_O)_2_]
*M* *_r_* = 988.65
Tetragonal, 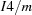

*a* = 14.8313 (10) Å
*c* = 9.3966 (8) Å
*V* = 2066.9 (2) Å^3^
*Z* = 2
Mo *K*α radiation
μ = 3.95 mm^−1^
*T* = 150 K
0.37 × 0.27 × 0.14 mm

### Data collection  

Bruker APEXII diffractometer
Absorption correction: multi-scan (*SADABS*; Bruker, 2006 ▸) *T*_min_ = 0.409, *T*_max_ = 0.575
4471 measured reflections
1248 independent reflections
940 reflections with *I* > 2σ(*I*)
*R*_int_ = 0.034

### Refinement  

*R*[*F* ^2^ > 2σ(*F* ^2^)] = 0.031
*wR*(*F* ^2^) = 0.073
*S* = 1.06
1248 reflections
81 parameters
H atoms treated by a mixture of independent and constrained refinement
Δρ_max_ = 0.50 e Å^−3^
Δρ_min_ = −0.34 e Å^−3^

### 

Data collection: *APEX2* (Bruker, 2006 ▸); cell refinement: *SAINT* (Bruker, 2006 ▸); data reduction: *SAINT*; program(s) used to solve structure: *SIR97* (Altomare *et al.*, 1999 ▸); program(s) used to refine structure: *SHELXL97* (Sheldrick, 2008 ▸); molecular graphics: *ORTEP-3 for Windows* (Farrugia, 2012 ▸); software used to prepare material for publication: *WinGX* (Farrugia, 2012 ▸).

## Supplementary Material

Crystal structure: contains datablock(s) I, New_Global_Publ_Block. DOI: 10.1107/S2056989015003722/wm5127sup1.cif

Structure factors: contains datablock(s) I. DOI: 10.1107/S2056989015003722/wm5127Isup2.hkl

**Fig. 1.**
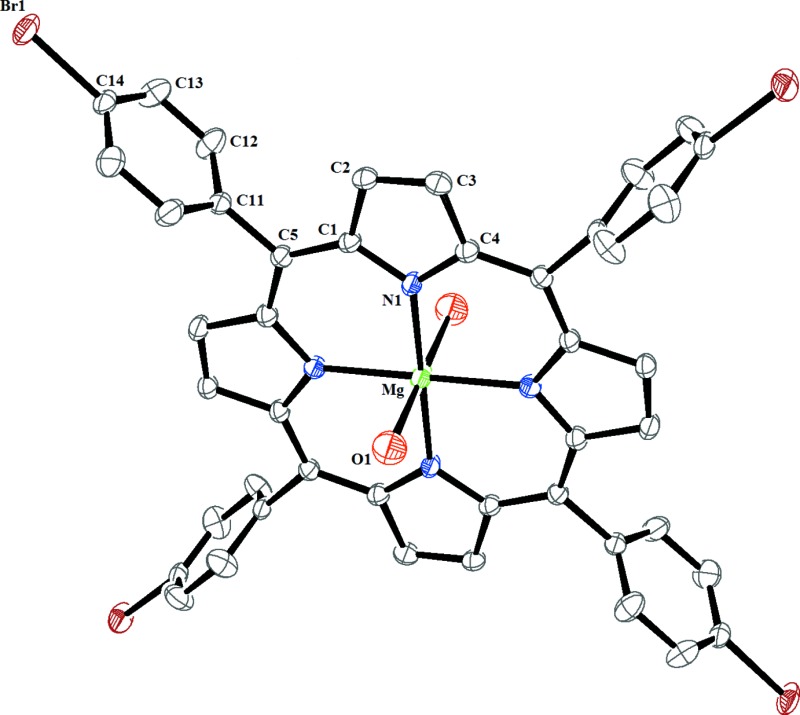
A view of the molecular structure of [Mg(C44H24N4Br4)(H2O)2] showing the atom numbering scheme. Displacement ellipsoids are drawn at the 50% probability level. H atoms have been omitted for clarity.

44 24 4 4 2 2 . DOI: 10.1107/S2056989015003722/wm5127fig1.tif
A view of the mol­ecular structure of [Mg(C_44_H_24_N_4_Br_4_)(H_2_O)_2_] showing the atom numbering scheme. Displacement ellipsoids are drawn at the 50% probability level. H atoms have been omitted for clarity.

**Fig. 2.**
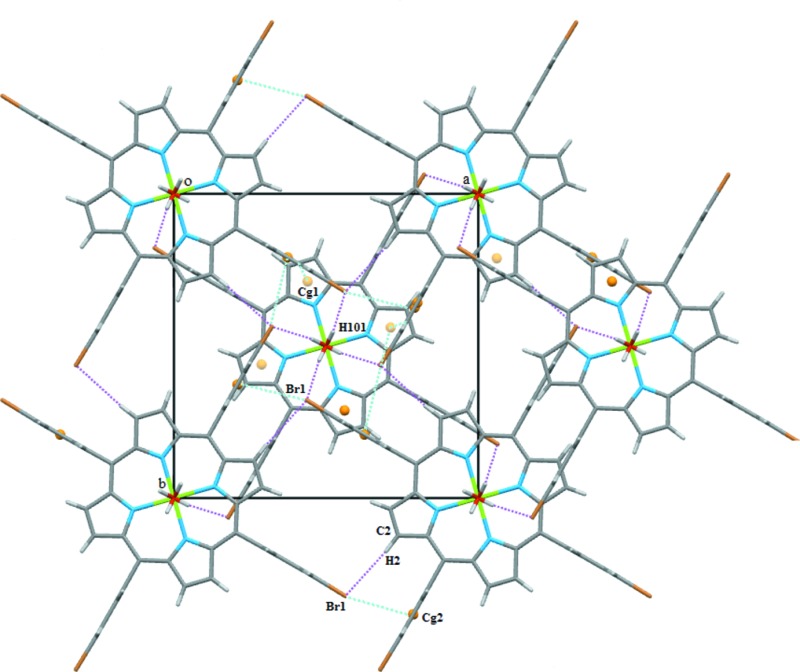
A portion of the crystal packing of the title complex, viewed down [001], showing C—H···π interactions (dotted light-blue lines) and C—H···Br and O—H···Br hydrogen bonds (dashed pink lines).

. DOI: 10.1107/S2056989015003722/wm5127fig2.tif
A portion of the crystal packing of the title complex, viewed down [001], showing C—H⋯π inter­actions (dotted light-blue lines) and C—H⋯Br and O—H⋯Br hydrogen bonds (dashed pink lines).

CCDC reference: 1050856

Additional supporting information:  crystallographic information; 3D view; checkCIF report

## Figures and Tables

**Table 1:** Hydrogen-bond geometry (, ) *Cg*1 is the centroid of the N1/C1C4 pyrrole ring.

*D*H*A*	*D*H	H*A*	*D* *A*	*D*H*A*
C13H13*Cg*1^i^	0.95	2.74	3.615(3)	153
O1H1*O*1Br1^ii^	1.10(5)	2.69(5)	3.741(3)	159(4)
C2H2Br1^iii^	0.95	2.97	3.914(3)	175(1)

Symmetry codes: (i) 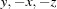; (ii) 
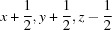
; (iii) 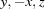.

## supplementary crystallographic information

### S1. Synthesis and crystallization

To a solution of [Mg(TBrPP)] (15 mg, 0.015 mmol) in di­chloro­methane (10 ml) was added an excess of di­methyl­glyoxime (50 mg, 0.431 mmol). The reaction mixture was stirred at room temperature and at the end of the reaction, the color of the solution gradually changed from purple to blue–purple. Black coloured crystals of the title complex were obtained by diffusion of *n*-hexane through the di­chloro­methane solution. The two water molecules coordinating to the magnesium atom most probably originated from the undistilled di­chloro­methane solvent.

### S2. Refinement

All H atoms attached to C atoms were fixed geometrically and treated as riding with C—H = 0.95 Å and *U*_iso_(H) = 1.2*U*_eq_(C). The H atom position of the axially bonded aqua ligand was calculated with the CALC-OH program (Nardelli, 1999). The molecular symmetry of the water molecule is not compatible with the fourfold axis, hence the occupancy of this H atom was fixed to 0.5.

### Crystal data

**Table:**

[Mg(C_44_H_24_Br_4_N_4_)(H_2_O)_2_]	*D*_x_ = 1.589 Mg m^−^^3^
*M**_r_* = 988.65	Mo *K*α radiation, λ = 0.71073 Å
Tetragonal, *I*4/*m*	Cell parameters from 1411 reflections
Hall symbol: -I 4	θ = 2.6–26.9°
*a* = 14.8313 (10) Å	µ = 3.95 mm^−^^1^
*c* = 9.3966 (8) Å	*T* = 150 K
*V* = 2066.9 (2) Å^3^	Prism, black
*Z* = 2	0.37 × 0.27 × 0.14 mm
*F*(000) = 976	

### Data collection

**Table:**

Bruker APEXII diffractometer	940 reflections with *I* > 2σ(*I*)
Graphite monochromator	*R*_int_ = 0.034
CCD rotation images, thin slices scans	θ_max_ = 27.5°, θ_min_ = 3.8°
Absorption correction: multi-scan (*SADABS*; Bruker, 2006)	*h* = −14→19
*T*_min_ = 0.409, *T*_max_ = 0.575	*k* = −18→18
4471 measured reflections	*l* = −5→12
1248 independent reflections	

### Refinement

**Table:**

Refinement on *F*^2^	Primary atom site location: structure-invariant direct methods
Least-squares matrix: full	Secondary atom site location: difference Fourier map
*R*[*F*^2^ > 2σ(*F*^2^)] = 0.031	Hydrogen site location: inferred from neighbouring sites
*wR*(*F*^2^) = 0.073	H atoms treated by a mixture of independent and constrained refinement
*S* = 1.06	*w* = 1/[σ^2^(*F*_o_^2^) + (0.0364*P*)^2^ + 0.9155*P*] where *P* = (*F*_o_^2^ + 2*F*_c_^2^)/3
1248 reflections	(Δ/σ)_max_ = 0.009
81 parameters	Δρ_max_ = 0.50 e Å^−^^3^
0 restraints	Δρ_min_ = −0.34 e Å^−^^3^

### Special details

**Table:**

Geometry. All s.u.'s (except the s.u. in the dihedral angle between two l.s. planes) are estimated using the full covariance matrix. The cell s.u.'s are taken into account individually in the estimation of s.u.'s in distances, angles and torsion angles; correlations between s.u.'s in cell parameters are only used when they are defined by crystal symmetry. An approximate (isotropic) treatment of cell s.u.'s is used for estimating s.u.'s involving l.s. planes.
Refinement. Refinement of *F*^2^ against ALL reflections. The weighted *R*-factor *wR* and goodness of fit *S* are based on *F*^2^, conventional *R*-factors *R* are based on *F*, with *F* set to zero for negative *F*^2^. The threshold expression of *F*^2^ > 2σ(*F*^2^) is used only for calculating *R*-factors(gt) *etc*. and is not relevant to the choice of reflections for refinement. *R*-factors based on *F*^2^ are statistically about twice as large as those based on *F*, and *R*- factors based on ALL data will be even larger.

### Fractional atomic coordinates and isotropic or equivalent isotropic displacement parameters (Å^2^)

**Table:**

	*x*	*y*	*z*	*U*_iso_*/*U*_eq_	Occ. (<1)
Mg	0.5	0.5	0.5	0.0248 (5)	
O1	0.5	0.5	0.2636 (4)	0.0376 (9)	
H1O1	0.473 (4)	0.560 (3)	0.210 (6)	0.045*	0.5
N1	0.46158 (17)	0.36592 (17)	0.5	0.0192 (6)	
C1	0.3751 (2)	0.3337 (2)	0.5	0.0183 (7)	
C2	0.3771 (2)	0.2366 (2)	0.5	0.0195 (7)	
H2	0.3262	0.1977	0.5	0.023*	
C3	0.4643 (2)	0.2109 (2)	0.5	0.0219 (7)	
H3	0.4865	0.1508	0.5	0.026*	
C4	0.5177 (2)	0.2931 (2)	0.5	0.0196 (7)	
C5	0.2966 (2)	0.3872 (2)	0.5	0.0179 (7)	
C11	0.2081 (2)	0.33768 (19)	0.5	0.0192 (7)	
C12	0.16663 (16)	0.31403 (16)	0.6263 (3)	0.0307 (6)	
H12	0.1942	0.3299	0.714	0.037*	
C13	0.08517 (16)	0.26732 (17)	0.6273 (3)	0.0324 (6)	
H13	0.0572	0.2512	0.7146	0.039*	
C14	0.0463 (2)	0.2451 (2)	0.5	0.0229 (7)	
Br1	−0.06342 (2)	0.17805 (2)	0.5	0.03531 (15)	

### Atomic displacement parameters (Å^2^)

**Table:**

	*U*^11^	*U*^22^	*U*^33^	*U*^12^	*U*^13^	*U*^23^
Mg	0.0142 (7)	0.0142 (7)	0.0461 (16)	0	0	0
O1	0.0376 (13)	0.0376 (13)	0.038 (2)	0	0	0
N1	0.0124 (13)	0.0162 (14)	0.0290 (16)	−0.0007 (10)	0	0
C1	0.0169 (16)	0.0150 (16)	0.0231 (18)	−0.0024 (12)	0	0
C2	0.0199 (16)	0.0152 (15)	0.0233 (18)	−0.0031 (12)	0	0
C3	0.0246 (17)	0.0137 (15)	0.0274 (19)	0.0007 (13)	0	0
C4	0.0186 (16)	0.0167 (16)	0.0234 (18)	−0.0014 (13)	0	0
C5	0.0164 (16)	0.0165 (16)	0.0209 (18)	−0.0009 (13)	0	0
C11	0.0166 (16)	0.0114 (15)	0.0296 (19)	0.0002 (12)	0	0
C12	0.0260 (13)	0.0389 (15)	0.0272 (14)	−0.0117 (11)	0.0002 (11)	−0.0054 (12)
C13	0.0295 (14)	0.0356 (15)	0.0321 (15)	−0.0096 (11)	0.0095 (12)	−0.0013 (12)
C14	0.0158 (16)	0.0129 (16)	0.040 (2)	−0.0010 (12)	0	0
Br1	0.01523 (19)	0.0223 (2)	0.0684 (3)	−0.00419 (14)	0	0

### Geometric parameters (Å, º)

**Table:**

Mg—N1^i^	2.069 (2)	C3—C4	1.454 (4)
Mg—N1	2.069 (2)	C3—H3	0.95
Mg—N1^ii^	2.069 (2)	C4—C5^iii^	1.412 (4)
Mg—N1^iii^	2.069 (2)	C5—C4^i^	1.412 (4)
Mg—O1^ii^	2.221 (4)	C5—C11	1.504 (4)
Mg—O1	2.221 (4)	C11—C12	1.382 (3)
O1—H1O1	1.10 (5)	C11—C12^iv^	1.382 (3)
N1—C4	1.363 (4)	C12—C13	1.393 (3)
N1—C1	1.368 (4)	C12—H12	0.95
C1—C5	1.409 (4)	C13—C14	1.369 (3)
C1—C2	1.440 (4)	C13—H13	0.95
C2—C3	1.348 (4)	C14—C13^iv^	1.369 (3)
C2—H2	0.95	C14—Br1	1.906 (3)
			
N1^i^—Mg—N1	89.998 (2)	N1—C4—C3	109.4 (3)
N1—Mg—N1^ii^	180.00 (14)	C5^iii^—C4—C3	125.1 (3)
O1^ii^—Mg—O1	180	C1—C5—C4^i^	126.4 (3)
Mg—O1—H1O1	117 (3)	C1—C5—C11	116.5 (3)
C4—N1—C1	107.1 (2)	C4^i^—C5—C11	117.1 (3)
C4—N1—Mg	126.4 (2)	C12—C11—C12^iv^	118.3 (3)
C1—N1—Mg	126.5 (2)	C12—C11—C5	120.85 (15)
N1—C1—C5	125.3 (3)	C12^iv^—C11—C5	120.85 (15)
N1—C1—C2	109.3 (3)	C11—C12—C13	121.3 (2)
C5—C1—C2	125.5 (3)	C11—C12—H12	119.4
C3—C2—C1	107.6 (3)	C13—C12—H12	119.4
C3—C2—H2	126.2	C14—C13—C12	118.7 (2)
C1—C2—H2	126.2	C14—C13—H13	120.7
C2—C3—C4	106.6 (3)	C12—C13—H13	120.7
C2—C3—H3	126.7	C13—C14—C13^iv^	121.9 (3)
C4—C3—H3	126.7	C13—C14—Br1	119.04 (15)
N1—C4—C5^iii^	125.5 (3)	C13^iv^—C14—Br1	119.05 (15)
			
N1^i^—Mg—N1—C4	180	C1—N1—C4—C3	0
N1^iii^—Mg—N1—C4	0	Mg—N1—C4—C3	180
O1^ii^—Mg—N1—C4	−90	C2—C3—C4—N1	0
O1—Mg—N1—C4	90	C2—C3—C4—C5^iii^	180
N1^i^—Mg—N1—C1	0	N1—C1—C5—C4^i^	0
N1^iii^—Mg—N1—C1	180	C2—C1—C5—C4^i^	180
O1^ii^—Mg—N1—C1	90	N1—C1—C5—C11	180
O1—Mg—N1—C1	−90	C2—C1—C5—C11	0
C4—N1—C1—C5	180	C1—C5—C11—C12	89.7 (3)
Mg—N1—C1—C5	0	C4^i^—C5—C11—C12	−90.3 (3)
C4—N1—C1—C2	0	C1—C5—C11—C12^iv^	−89.7 (3)
Mg—N1—C1—C2	180	C4^i^—C5—C11—C12^iv^	90.3 (3)
N1—C1—C2—C3	0	C12^iv^—C11—C12—C13	0.2 (5)
C5—C1—C2—C3	180	C5—C11—C12—C13	−179.3 (2)
C1—C2—C3—C4	0	C11—C12—C13—C14	−0.1 (4)
C1—N1—C4—C5^iii^	180	C12—C13—C14—C13^iv^	0.1 (5)
Mg—N1—C4—C5^iii^	0	C12—C13—C14—Br1	178.2 (2)

Symmetry codes: (i) *y*, −*x*+1, −*z*+1; (ii) −*x*+1, −*y*+1, −*z*+1; (iii) −*y*+1, *x*, *z*; (iv) *x*, *y*, −*z*+1.

### Hydrogen-bond geometry (Å, º)

Cg1 is the centroid of the N1/C1–C4 pyrrole ring.

**Table:**

*D*—H···*A*	*D*—H	H···*A*	*D*···*A*	*D*—H···*A*
C13—H13···*Cg*1^v^	0.95	2.74	3.615 (3)	153
O1—H1*O*1···Br1^vi^	1.10 (5)	2.69 (5)	3.741 (3)	159 (4)
C2—H2···Br1^vii^	0.95	2.97	3.914 (3)	175 (1)

Symmetry codes: (v) *y*, −*x*, −*z*; (vi) *x*+1/2, *y*+1/2, *z*−1/2; (vii) *y*, −*x*, *z*.
