# Supplementary figures and images for: Crystal structure of di­aqua­[5,10,15,20-tetra­kis­(4-bromo­phen­yl)porphyrinato-κ4 N]magnesium
Source: Acta Crystallogr E Crystallogr Commun. 2015 Feb 28;71(Pt 3):m73–4. doi: 10.1107/S2056989015003722 (PMC4350761; doi:10.1107/S2056989015003722)

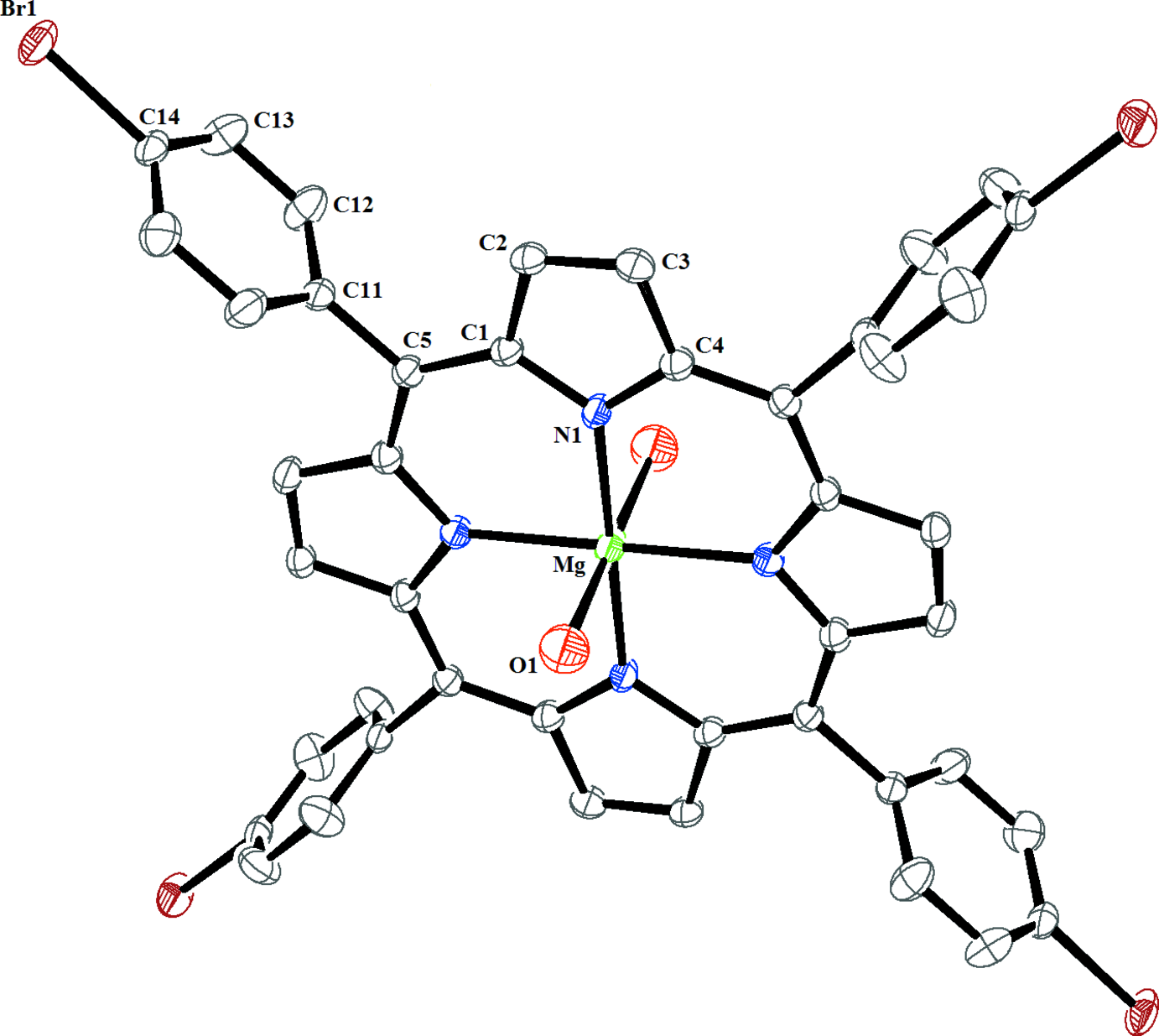

Supplement: Supplementary file 3 [file e-71-00m73-fig1.tif]

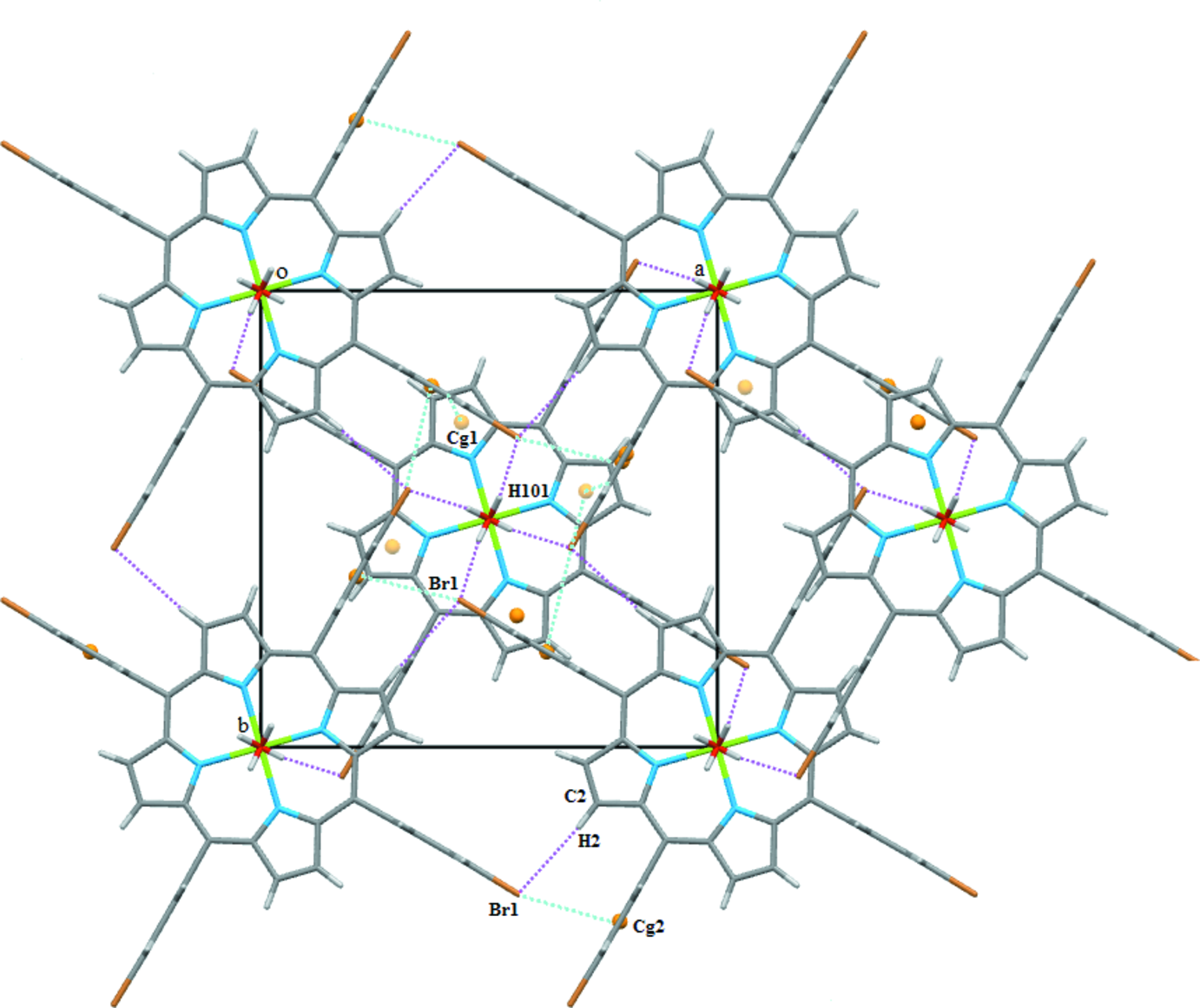

Supplement: Supplementary file 4 [file e-71-00m73-fig2.tif]
